# 3D-printed porous zinc scaffold combined with bioactive serum exosomes promotes bone defect repair in rabbit radius

**DOI:** 10.18632/aging.205891

**Published:** 2024-05-31

**Authors:** Baoxin Zhang, Zhiwei Pei, Wanxiong He, Wei Feng, Ting Hao, Mingqi Sun, Xiaolong Yang, Xing Wang, Xiangyu Kong, Jiale Chang, Guanghui Liu, Rui Bai, Chang Wang, Feng Zheng

**Affiliations:** 1 Department of Orthopedic Surgery, Suzhou Medical College of Soochow University, Suzhou 215000, Jiangsu, China; 2 Department of Orthopedic Surgery, Qinghai Provincial People’s Hospital, Xining 810000, Qinghai, China; 3 Graduate School of Inner Mongolia Medical University, Hohhot 010050, China; 4 Department of Orthopedic Surgery, The Second Affiliated Hospital of Inner Mongolia Medical University, Hohhot 010050, China; 5 Department of Orthopedic Surgery, Bayannur City Hospital, Bayannur 015000, China; 6 Department of Biomaterials Research Center, Shaanxi Key Laboratory of Biomedical Metallic Materials, Northwest Institute for Non-ferrous Metal Research, Shaanxi 710016, Xi’an, China

**Keywords:** bone defect, zinc scaffold, 3D printing, exosome, bone repair

## Abstract

Currently, the repair of large bone defects still faces numerous challenges, with the most crucial being the lack of large bone grafts with good osteogenic properties. In this study, a novel bone repair implant (degradable porous zinc scaffold/BF Exo composite implant) was developed by utilizing laser melting rapid prototyping 3D printing technology to fabricate a porous zinc scaffold, combining it under vacuum conditions with highly bioactive serum exosomes (BF EXO) and Poloxamer 407 thermosensitive hydrogel. The electron microscope revealed the presence of tea saucer-shaped exosomes with a double-layered membrane structure, ranging in diameter from 30–150 nm, with an average size of 86.3 nm and a concentration of 3.28E+09 particles/mL. *In vitro* experiments demonstrated that the zinc scaffold displayed no significant cytotoxicity, and loading exosomes enhanced the zinc scaffold’s ability to promote osteogenic cell activity while inhibiting osteoclast activity. *In vivo* experiments on rabbits indicated that the hepatic and renal toxicity of the zinc scaffold decreased over time, and the loading of exosomes alleviated the hepatic and renal toxic effects of the zinc scaffold. Throughout various stages of repairing radial bone defects in rabbits, loading exosomes reinforced the zinc scaffold’s capacity to enhance osteogenic cell activity, suppress osteoclast activity, and promote angiogenesis. This effect may be attributed to BF Exo’s regulation of p38/STAT1 signaling. This study signifies that the combined treatment of degradable porous zinc scaffolds and BF Exo is an effective and biocompatible strategy for bone defect repair therapy.

## INTRODUCTION

Severe bone defects caused by trauma, disease, or tumor resection often exceed the bone's natural healing capacity and thus require surgical repair and reconstruction [1]. Prolonged non-healing of these bone defects can lead to physical disability, resulting in the loss of individual work capacity and imposing a significant economic burden on both the individual and society [2]. Therefore, the treatment of bone defects is particularly crucial. Currently, common clinical methods for bone defect repair include autologous and allogeneic bone transplantation, as well as vascularized bone grafting [3]. However, challenges such as limited transplant donors, disease transmission, and susceptibility to infection still exist. Consequently, artificial biomaterials have been developed for bone defect reconstruction due to their accessibility and good formability [3–5]. In particular, additive manufacturing technology has been successfully used to produce non-biodegradable porous scaffolds, such as stainless steel, titanium, and tantalum, which possess high compressive strength and excellent fatigue resistance [6]. Nevertheless, the development of these implants is constrained by the potential need for secondary surgery and the possibility of releasing harmful metal ions. Additionally, non-biodegradable scaffolds fulfill both mechanical and biological functions, but they do not create additional space for new bone growth [7].

Through the research on biodegradable metal bone implants, it has been found that metallic elements such as zinc (Zn) can be used to manufacture orthopedic implants, gaining excellent mechanical properties [8]. Previous studies have shown that the degradation of zinc metals in living organisms can release Zn^2+^, which is beneficial for bone formation and can enhance the osteoinductivity of the scaffolds [2, 9]. In particular, zinc is one of the essential trace elements, and zinc ions can stimulate the proliferation and differentiation of osteoblasts by upregulating the expression of runt-related transcription factor 2 (Runx2) and the activity of alkaline phosphatase (ALP) [10]. Additionally, due to their unique degradation behavior, mechanical strength, and absence of hydrogen gas production, zinc and zinc-based alloys have greater potential as porous scaffolds compared to magnesium-based alloys [3, 7]. Notably, zinc is an essential element that possesses antioxidant and anti-inflammatory properties by regulating the formation of free radicals in the body [11–13]. However, the preparation techniques for zinc-containing polymers are diverse. For instance, electrospinning technology can mimic the extracellular matrix structure to promote cell proliferation, but the mechanical performance of the scaffold is very low [14, 15]. In contrast, freeze-drying and sintering techniques can produce scaffolds with good mechanical properties and porous structures, but the structure is not controllable [16]. To address the above issues, we propose the effective preparation of structurally controllable scaffolds using laser melting rapid prototyping technology [4]. Among these, porous zinc scaffolds are widely regarded as high-quality materials for bone repair due to their degradability, anti-inflammatory and antioxidant properties, excellent antibacterial capabilities, and osteoinductivity [17, 18]. However, the effectiveness of zinc scaffolds for bone defect repair is limited. It is crucial to improve their efficacy by combining them with other materials that support bone regeneration. Magnesium ions play multifaceted roles in osteogenic differentiation, serving not only as a component of bone mineral but also influencing bone formation and maintenance through various biochemical and molecular mechanisms. Magnesium ions contribute to the formation of new bone and the remodeling of old bone [1, 5].

In the field of tissue engineering, the three fundamental necessary elements were originally defined as biological material scaffolds, growth factors, and stem cells [19, 20]. However, with the advancement of tissue engineering, the use of stem cell-derived small extracellular vesicles (EVs) has been developed as a basis for cell-free regenerative medicine, rather than directly applying stem cells [21, 22]. As cell-free therapies gain prominence, the previously established three-element model has been simplified to two: scaffolds and bioactive components. Among the bioactive components, EVs have emerged as the most promising candidates for clinical application due to their exceptional bioactivity, stability, and highly feasible modular customized modifications [23]. Among these, exosomes, a representative type of small extracellular vesicles, are selective 30–150 nm vesicles that encapsulate proteins and nucleic acids, and have been increasingly utilized in tissue regeneration over the past few years [24, 25]. High bioactivity serum exosomes, in particular, have been demonstrated as a composite that promotes bone regeneration, and their combination with degradable metal scaffolds not only facilitates rapid bone regeneration, but also addresses the issue of mechanical support deficiency at the bone defect site [26, 27]. In our study, we utilized a laser melting rapid prototyping 3D printing technique to fabricate a porous zinc scaffold, combined under vacuum conditions with high bioactivity serum exosomes and poloxamer 407 thermosensitive hydrogel, resulting in a novel bone repair implant. As designed, this scaffold features a multi-level porous structure mimicking trabecular bone, with a Young's modulus (180.26 MPa) and compressive strength (10.1 MPa) close to that of trabecular bone.

In this study, we mixed Poloxamer 407 solution with prepared highly bioactive serum exosomes in a 9:1 ratio and then immersed the biodegradable zinc scaffold in the mixture at 4°C. Under vacuum conditions, we completely filled the scaffold with BF Exo gel composite. We used *in vitro* and *in vivo* studies to evaluate their potential to promote regeneration of radial bone defects in rabbits. Therefore, this study not only established the foundation for research on zinc and zinc-containing polymers in bone repair mechanisms, but also laid the groundwork for clinical applications of zinc-containing biomaterials combined with exosomes.

## MATERIALS AND METHODS

### Design and fabrication of porous zinc scaffold using 3D printing

The porous zinc scaffold with a dodecahedral porous structure was designed using Pro/Engineer (Pro/E) software and converted to an STL file format. The resulting STL file was imported into Materialise Magics 21.0 software for adding support, slicing, and applying SLM printing parameters, and then exported as a CLI file. Subsequently, the CLI file was imported into the HBD-80D selective laser melting rapid prototyping system (China) for fabrication, which consists of a fiber laser, scanning mirrors, powder deposition wall, precision positioning substrate, and control software. All fabrication processes were conducted under a high-purity argon gas environment, and the raw material used was industrial-grade spherical pure zinc powder (with a purity of 99.9% and a particle size range of 15–53 μm).

### Cell cytotoxicity assay for scaffolds

#### Preparation of scaffold leach solution

The extract was processed following the International Standard Operating Procedure (ISO 10993-5). Initially, the degradable 3D-printed porous zinc scaffold was placed in a 15 ml centrifuge tube and soaked in 75% alcohol for 30 minutes. After repeated washing with sterile PBS, the scaffold was incubated in MEM medium at a ratio of 6 cm^2^/ml at 37°C. After 72 hours, the culture medium was removed and stored at 4°C.

#### Cell culture

The L-929 fibroblast cells (Wuhan PunoCell, China, CL-0137) were cultured in Minimum Essential Medium (MEM) with 10% fetal bovine serum (Wuhan PunoCell, PM150410) and placed in a cell culture incubator at 37°C with 5% CO_2_. The culture medium contained 100 U/ml penicillin-streptomycin solution. Cell counting was performed using a hemocytometer, and the cells were seeded into a 96-well plate at a density of 5 × 10^3^ cells per well. Three wells were used for each group, and 100 μl of MEM culture medium was added to each well before incubating them in a 37°C, 5% CO_2_ incubator for 4 hours. The cells were then subjected to different treatments according to the following groups: (1) Control group, where normal MEM complete culture medium was added; (2) Zinc scaffold group, where the culture medium was replaced with a solution of the extract from the degradable porous scaffold (containing 10% fetal bovine serum). Both groups of cells were then placed in a culture incubator at 37°C with 5% CO_2_ and further cultured for 24, 48, and 72 hours.

#### Detection of cytotoxicity by MTT assay

At each time point of 24, 48, and 72 hours, a culture plate was removed, and MTT solution was added to each well. The plates were then placed in a cell culture incubator for an additional 4 hours. Subsequently, the culture medium was removed, and DMSO was added to each well. The mixture was then transferred to a new 96-well plate after thorough mixing. The liquid after dissolution was tested for absorbance values at 490 nm wavelength using an enzyme-linked immunosorbent assay reader (Thermo Fisher Scientific, USA, Multiskan 51119000). The absorbance values of the liquid in each well of the 96-well plate were measured. Relative cell viability (%) was calculated using the formula: Relative cell viability (%) = OD490’/avg (OD490C’) × 100%. Where, OD490' denotes the absorbance value of the experimental group minus the absorbance value of the blank control group, and avg (OD490C') represents the corrected average absorbance value of the control group.

### Extraction and characterization of high bioactive serum exosomes from rabbits

#### Extraction of high bioactive serum exosomes from rabbits

In the rabbit model of femoral fracture (21 days after modeling), blood was collected from the marginal ear vein and centrifuged to obtain serum. The serum was transferred to a new centrifuge tube and centrifuged at 4°C at 2000 g for 30 min to remove cell debris. The supernatant was then transferred to another centrifuge tube and centrifuged again at 4°C at 10,000 g for 45 min to remove larger vesicles. The filtrate was collected using a 0.45 μm filter membrane. Subsequently, the filtrate was transferred to a new centrifuge tube and centrifuged at 4°C at 100,000 g for 70 min. After removing the supernatant, the pellet was resuspended in 10 mL of pre-cooled 1 × PBS and centrifuged at 4°C at 100,000 g for 70 min to obtain Exosomes (Exos). After removing the supernatant, 20 μL of Exos were used for electron microscope examination, 10 μL were used for particle size analysis, and the remaining Exos were stored at −80°C.

#### Characterization of high bioactive serum exosomes from rabbits

First, 10 μL of extracellular vesicles were diluted to 30 μL and analyzed for size and concentration using a NanoFCM N30E nanoparticle analyzer. Then, 10 μL of the extracellular vesicle samples were magnified 60,000 times using an 80–120 KV transmission electron microscope (HT-7700, Hitachi, Japan) to observe their morphology and size, and photographs were taken. Additionally, extracellular vesicle proteins were extracted and their total concentration was determined using the BCA method (Shanghai Beyotime, China, P0010), followed by chemiluminescence using the Tanon ECL kit (Shanghai Tanon, China, 180-5001). The chemiluminescent images were captured and analyzed using the Tanon 5200 chemiluminescence imaging system following the instructions provided with the kit.

### Preparation and *in vitro* osteogenic performance evaluation of zinc scaffold/BF Exo composite implant

#### Preparation of zinc scaffold/BF Exo composite implant

Mix 25% by mass of Poloxamer 407 powder (Beijing Solarbio Technology Co., Ltd., China, S7071) into sterile phosphate-buffered saline (PBS), and refrigerate overnight at 4°C to obtain a clear solution. Then, mix this solution with prepared BF Exo at a 9:1 ratio, and immerse the zinc scaffold in the liquid under 4°C conditions. Lastly, vacuum fill the BF Exo gel composite into the scaffold for future use.

#### Physicochemical properties of zinc scaffold/BF Exo composite implants

The surface morphology and elemental analysis of the scaffold were imaged using SEM (Hitachi, S520) and X-ray diffractometer (Bruker, Germany, D8 Advance). Once the degradable porous zinc scaffold/BF Exo composite implant was prepared, it was immediately placed in a pre-cooled 2.5% glutaraldehyde solution and fixed overnight at 4°C. The solution was then replaced with 1% osmium tetroxide, and the sample was fixed for 2 hours at 4°C followed by three washes in 1 × PBS. The electron microscope samples were dehydrated with an ethanol gradient, dried at the critical point, and mounted on a specimen stage for three-dimensional morphology observation and analysis. Additionally, the X-ray diffractometer (Bruker, D8 Advance) was used to analyze the diffraction patterns of the degradable porous zinc scaffold/BF Exo composite implant.

#### Experiments on the Exos and zinc ion release from zinc scaffold/BF Exo composite implants

The Zinc scaffold and Zinc scaffold/BF Exo composite implant were placed in separate 1.5 ml centrifuge tubes, followed by the addition of 1 ml sterile PBS to each tube. The tubes were then incubated at 37°C for 1, 3, 5, and 7 days, after which the supernatant was collected. Utilizing the BCA protein concentration assay kit (Shanghai Biyuntian, China, P0010) in conjunction with a microplate reader (Thermo Fisher Scientific, Multiskan FC) to measure the OD values at 562 nm, the protein concentration of the samples was calculated based on the standard curve and sample volume. Subsequently, the zinc ion concentration of the samples was determined by employing the zinc colorimetric assay kit (Wuhan Elisa, E-BC-K137-M) with measurements made at 560 nm using the microplate reader (Thermo Fisher Scientific, Multiskan FC), and calculations were carried out based on the standard curve and sample volume, as per the provided instructions.

#### Biological activity characterization of zinc scaffold/BF Exo composite implant in vitro

Place the prepared biodegradable porous zinc scaffold and zinc scaffold/BF Exo composite implants in a well plate. Bone marrow mesenchymal stem cells (BMSCs, rabbit-derived, primary culture) or mouse monocyte leukemia cells RAW264.7 (Wuhan Fenghui, China, CL0266) were seeded in the 48-well plate at a density of 1 × 10^4^ cells/well. The cells were then cultured in a CO_2_ incubator at 37°C for 3 days. Each cell group was assigned 4 replicate wells, and the groups were as follows: ① Group A: without exosome scaffolds; ② Group B: with exosome scaffolds.

Following the co-cultivation of zinc-degradable stent/BF Exo composite implants with BMSCs for 3 days, the culture medium was replaced with DMEM complete medium containing osteogenic induction fluid (10% FBS). Cell proliferation ability was assessed using the Cell Counting Kit-8 (CCK-8) assay (Glpbio, USA, GK10001) after induction for 1, 3, 5, and 7 days. Alkaline phosphatase staining was conducted using BCIP/NBT staining reagent (Shanghai Biyuntian, C3206) for continuous induction for 3 and 7 days, followed by incubation in the dark at room temperature and rinsing with distilled water. The stained cells were observed under an inverted microscope and photographed. After continuous induction for 14 and 21 days, the original culture medium was removed and the cells were fixed in 95% ethanol for 30–60 minutes. Staining with 1% Alizarin Red solution (Beijing Solarbio, G1452) was carried out for 30 minutes at room temperature. Following multiple washes with PBS, the stained cells were observed under an inverted microscope and photographed. Prior to these steps, dexamethasone (0.1 μM), ascorbic acid (0.2 mM), and β-glycerophosphate (10 mM) were combined in a serum-free culture medium, filtered through a 0.22 μm filter, and stored at 4°C for future use.

Furthermore, scaffolds were placed in the culture wells according to the grouping, and 50 ng/ml RANKL culture medium (Suzhou Novoprotein, China, CJ94) was added. RAW264.7 cells in each group were induced for osteoclastogenesis in a CO_2_ incubator at 37°C. Cell proliferation capability was assessed using the CCK-8 assay kit (Glpbio, GK10001) on days 1, 3, 5, and 7 post-induction. After 7 days of induction, cells were stained with the Acid Phosphatase Staining Kit (Beijing Solarbio, G1492) as per the manufacturer's instructions, washed, air-dried, and observed under a microscope for coloration, followed by photographic documentation.

### Implantation experiment of zinc scaffolds/BF Exo composite implants *in vivo*

#### Establishment of a rabbit radius defect model and implantation of composite implants

All animal procedures and experiments were approved by the ethics committee and carried out under aseptic surgical conditions to create a rabbit radial bone defect model. The animal experiments utilized New Zealand white rabbits (purchased from Jiangsu Zhenlin Biotechnology Co., Ltd., animal license number: SCXK (Su) 2016-0008), with the formal experiments commencing after a one-week acclimatization period.

After administering anesthesia, the rabbit's midshaft of the radius bone was exposed. The bone membrane was cut, and a 1.5 cm segment of the radial bone was removed using a bone cutter at the point of maximum curvature of the midshaft. The bone edges were then adjusted using bone forceps, followed by the swift implantation of the prepared graft into the radial bone defect. The bone membrane and skin were sutured, and penicillin was administered for infection prevention following disinfection. After the modeling, the animals were respectively maintained for 8 and 12 weeks before euthanasia, with subcutaneous injections of Alizarin Red S (30 mg/kg) and Calcein Green (10 mg/kg) given three days and ten days before euthanasia.

#### Final sampling

Animals were euthanized at 8 and 12 weeks after modeling, and blood, liver, kidney, and radius samples were collected from the rabbits. The collected blood was processed into serum and stored at −80°C for further testing, while the liver, kidney, and radius samples were fixed in 4% paraformaldehyde solution at room temperature.

#### The ELISA experiment for rabbit serum

Serum samples were obtained from New Zealand white rabbits at 8 and 12 weeks post-surgery to measure levels of bone formation markers (PICP and BALP), bone resorption marker (ICTP), and angiogenesis markers (VEGF, FGF2) using ELISA. The ELISA kits were brought to room temperature, standard solutions were prepared as per the instruction manual, and blank, standard, and sample wells were set up. Blank wells were not given any sample or enzyme reagent, while 50 μL of sample or standard solution and 100 μL of enzyme reagent were added to the remaining wells, and then incubated at 37°C for 1 hour. After washing the plate 5 times, a chromogenic reagent was added and incubated at 37°C for 15 minutes. Finally, the reaction was stopped by adding stop solution and absorbance values for each well were measured at a wavelength of 450 nm.

#### The fluorescence experiment of Alizarin Red S and Calcein Green from the rabbit radius bone

After fixing, decalcifying, and dehydrating the rabbit radius tissues, they are cooled according to embedding requirements. Once the wax blocks solidify, they are removed from the embedding molds and trimmed. The embedded wax blocks are then placed in a 4°C refrigerator to set, followed by removal and mounting on a microtome for cutting into 5 μm wax sections. The sections are flattened, lifted out of water using glass slides, air-dried, and then placed in a 37°C oven for drying. Subsequently, the fluorescent Alizarin Red S and Calcein Green-labeled rabbit radius tissues are observed and photographed using a laser confocal microscope (Shanghai Leica Instrument Co., Ltd., China, STELLARIS 5 Cryo).

#### The methylene blue-acid magenta staining experiment for rabbit radius bone

After fixing, decalcifying, and dehydrating rabbit radius tissues, the melted paraffin is poured into embedding molds. The tissues are removed from the dehydration chamber and placed into the embedding molds according to the embedding requirements, and labeled accordingly before the paraffin solidifies. The molds are then cooled on a −20°C freezing stage, and once the paraffin blocks have solidified, they are removed from the embedding molds and trimmed. The embedded paraffin blocks are then placed in a 4°C refrigerator to set before being cut into 5 μm paraffin sections. These sections are flattened and lifted from water using glass slides, and then air dried before being baked in a 37°C oven. Following baking, the sections are immediately stained with methylene blue-acid magenta, and then placed in a 37°C drying machine. After this, neutral resin is used to mount the slides and they are left to air dry at room temperature. Finally, the different rabbit radius tissue structures are observed and photographed under an optical microscope (Olympus BX51, Japan).

#### Experiment on HE staining of rabbit liver and kidney tissues

The Hematoxylin and Eosin (HE) staining kit (Shanghai Biyuntian Biotechnology, China, C0105M) was used to stain rabbit liver and kidney tissues. The tissues were dehydrated, embedded, sectioned, stained with HE, and coverslipped according to the instructions. Afterward, the tissue structures were observed and photographed under a light microscope.

#### The micro-CT imaging experiment on rabbit radius tissues

After fixation processing of all sampling specimens, original images were obtained by Micro-CT scanning (Bruker, Germany, SkyScan 1276). The NRecon 3D reconstruction software (software version V1.7.4.2, Bruker, Germany) was used to reconstruct selected areas of the original images, followed by analysis of the region of interest (ROI) using CT Analyser (software version 1.18.8.0, Bruker, Germany). The ROI, defined as the implanted material and the surrounding area with a diameter of 0.2 mm, was analyzed to detect the situation of new bone formation both inside the implanted material and in the surrounding area. After setting uniform parameters, the software calculated parameters such as the total volume of tissue (Tissue volume, TV), volume of new bone in the ROI (Bone volume, BV), bone volume fraction in the ROI (Bone volume/Tissue volume, BV/TV), bone mineral content (BMC), and bone mineral density (BMD) within the ROI region.

### Immunohistochemical testing

The protein expression levels of p38 and STAT1 in the bone tissue of each group of New Zealand white rabbits were detected by immunohistochemistry 12 weeks after surgery. After bone tissue sectioning, baking, dewaxing, and hydration, antigen retrieval was performed using citrate buffer. After blocking with 5% BSA, tissues were incubated with primary antibodies p38 (1:100) and STAT1 (1:100) overnight at 4°C, followed by secondary antibody incubation with HRP-conjugated goat anti-rabbit IgG (H+L) (1:100). The sections were then stained with DAB, counterstained with hematoxylin, dehydrated, cleared, and observed under a microscope.

### Statistical analysis

All experiments were repeated at least 3 times. Data were represented using mean ± standard deviation (*Mean ± SD*), and analyzed using GraphPad Prism 8. Student's *t*-test was used for comparing means of two groups and ANOVA test for comparing means of multiple groups. A significance level of *P* < 0.05 was used to determine statistical significance.

### Availability of data and materials

The original contributions presented in the study are included in the article material, further inquiries can be directed to the corresponding authors.

## RESULTS

### Preparation of 3D-printed porous zinc scaffold

**Figure 1:**
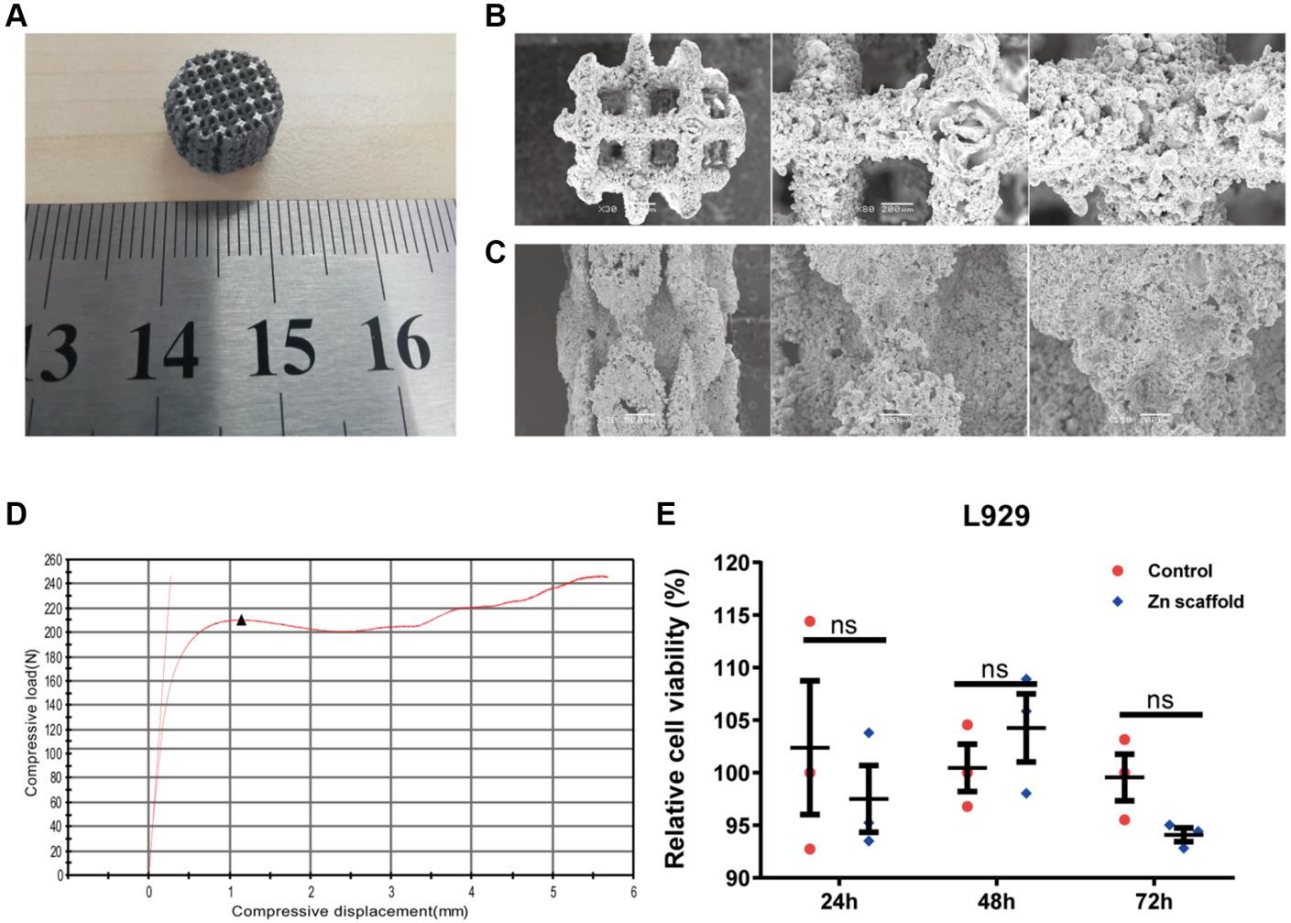
**Preparation of 3D-printed porous zinc scaffold.** (**A**) 3D-printed macroscopic image of a porous zinc scaffold; (**B**) SEM images of the porous zinc scaffold at 30, 80, and 150 times magnification in the frontal view; (**C**) SEM images of the porous zinc scaffold at 30, 80, and 150 times magnification in the side view; (**D**) Stress-strain plot of the 3D-printed porous zinc scaffold, with compressive displacement on the x-axis and compressive load on the y-axis, where the black triangles represent the first peak compressive load; (**E**) *In vitro* cytotoxicity evaluation of the 3D-printed porous zinc scaffold.

This study optimized the experimental parameters and established the 3D printing process parameters: laser power of 125 W, scanning speed of 1050 mm/s, scan spacing of 60 μm, and layer thickness of 30 μm. Porous samples of size Φ10 mm × 15 mm were prepared for *in vitro* experiments (Figure 1A). Previous research has indicated that in 3D-printed scaffolds, aperture sizes of 500–600 micrometers and a porosity of 60–70% are the optimal design parameters for promoting bone formation ^[4]^. Therefore, to ensure the scaffold possesses sufficient mechanical properties, the scaffold structure was designed based on the following structural parameters. The scaffold was designed as a porous structure with interconnected pores, an average aperture size of approximately 500 μm, and a porosity of approximately 70% (Figure 1B, 1C). Using an INSTRON universal testing machine (model: 3367), compression tests at room temperature were conducted on the radial bone-sized scaffold under controlled conditions of a compression rate of 0.5 mm/min and a compression variable of 40%, resulting in load-displacement curves, and the calculation of the scaffold's Young's modulus and compressive strength (Figure 1D). From the graph, it is evident that the maximum compression load is 210 N, the Young's modulus is 180.26 MPa, and the compressive strength is 10.1 MPa. Subsequently, the scaffold extraction liquid and L-929 cells were co-cultured for 24, 48, and 72 hours, followed by MTT experiments. The results indicate no significant differences in cell viability between the degradable 3D-printed porous zinc scaffold extraction group and the control group (Figure 1E). This proves that the degradable zinc scaffold exhibits no significant cytotoxicity and can be used in subsequent experiments.

### Extraction and identification of exosomes from rabbit serum for biological activity

**Figure 2:**
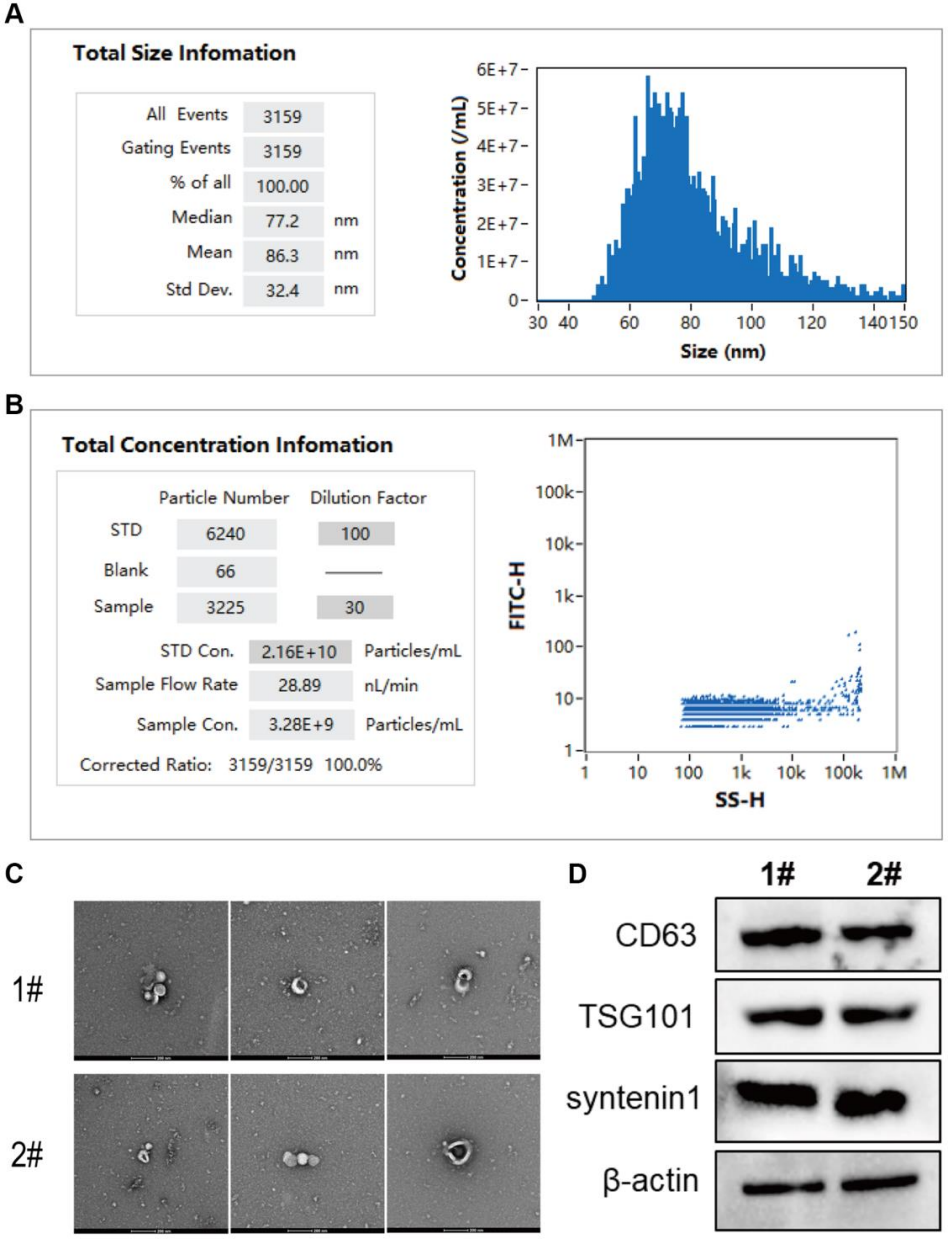
**Extraction and identification of exosomes from rabbit serum for biological activity.** (**A**) The analysis of exosome particle size using nanoscale flow cytometry; (**B**) The analysis of exosome concentration. (**C**, **D**) Transmission electron microscopy images of exosomes in serum samples from rabbit models with femoral fractures, where C shows the exosome suspension at dilutions of 10, 30, and 50-fold from left to right, and D illustrates the protein expression of CD63, TSG101, and syntenin1 in exosomes from the serum of rabbit models with femoral fractures.

In this study, high-activity serum exosomes were extracted from the ear vein blood of rabbits with femoral fractures (21 days after modeling) using ultracentrifugation. The exosome morphology was observed using transmission electron microscopy, and the nanoparticle size and concentration were measured using nanoscale flow cytometry. Initially, the serum exosomes from rabbits with femoral fractures were analyzed using a nanoparticle size and zeta potential analyzer (Figure 2A, 2B). The average particle size of the exosomes was determined to be 86.3 nm (Figure 2A), with a concentration of 3.28E+9 particles/mL (Figure 2B). Subsequently, the exosome suspension was diluted with PBS at 10, 30, and 50-fold ratios. Transmission electron microscopy analysis revealed that the size of the serum exosomes from the two samples was uniform, with clear and intact structures, displaying a typical vesicular morphology (Figure 2C). Furthermore, Western blot experiments were conducted on the serum exosomes from two rabbit models with femoral fractures, and the results showed the expression of CD63, TSG101, and syntenin1 proteins in the serum exosomes (Figure 2D and Supplementary Figure 1).

### Preparation and *in vitro* osteogenic performance assessment of zinc scaffold/BF Exo composite implant

**Figure 3:**
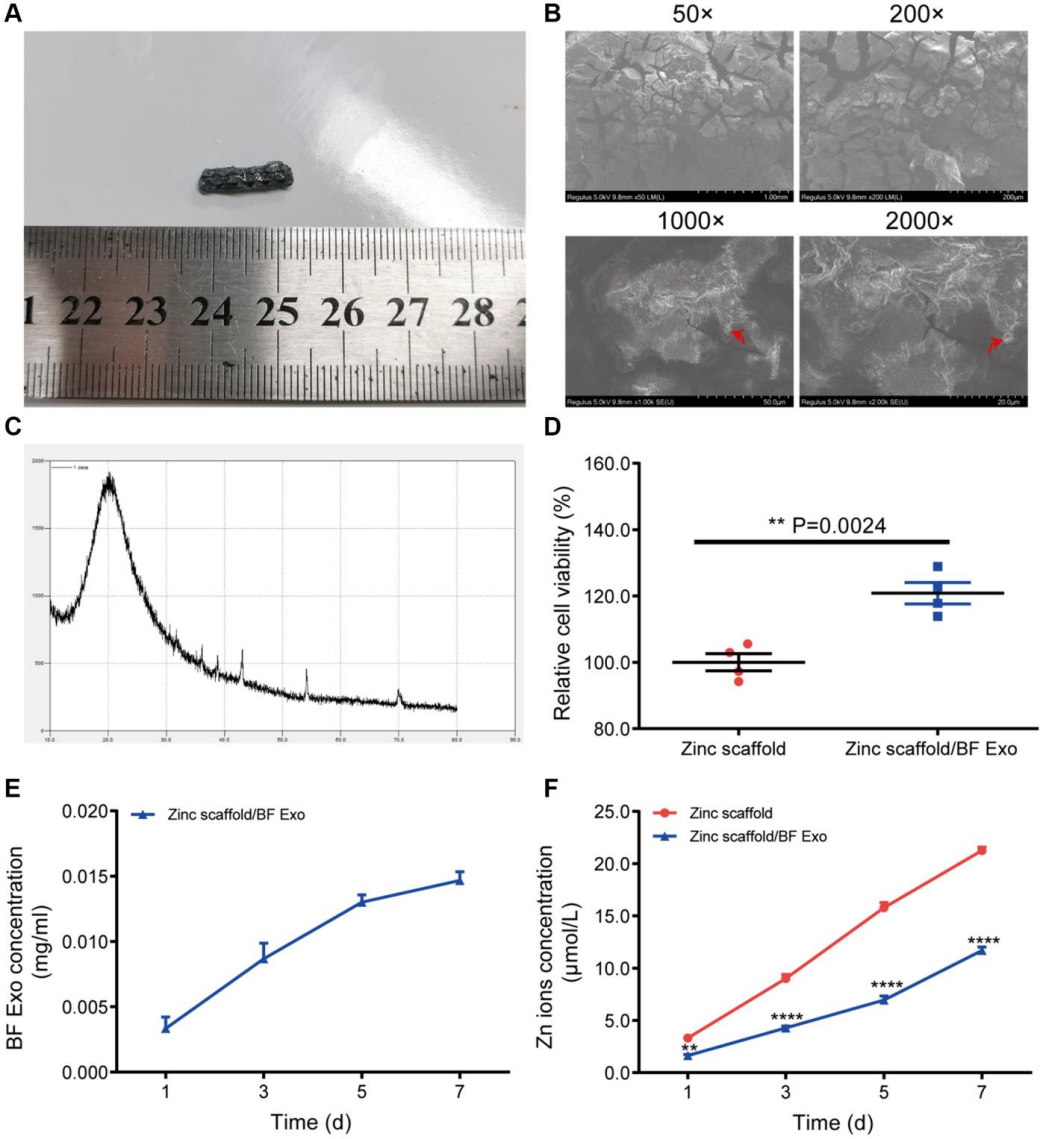
**Preparation of zinc scaffold/BF Exo composite implant.** (**A**) Solid images of degradable zinc stent/BF Exo composite implant; (**B**) Electron microscopy images of degradable zinc stent/BF Exo composite implant at 50, 200, 1000, and 2000 times magnification, with red arrows indicating small amounts of exosomes encapsulated in the gel; (**C**) X-ray diffraction patterns of degradable zinc stent/BF Exo composite implant; (**D**) CCK-8 experiment results after co-culturing BMSCs cells with degradable zinc stent/BF Exo composite implant for 3 days; (**E**) *In vitro* release results of exosomes from zinc stent/BF Exo composite implants after incubation for 1, 3, 5, and 7 days; (**F**) *In vitro* release results of zinc ions after incubating two types of stents for 1, 3, 5, and 7 days (^**^P < 0.01, ^****^P < 0.0001 vs. Zinc scaffold, *n* = 3).

The designed scaffold for *in vivo* experiments has an outer dimension of Φ3.5 mm × 15 mm for the radius bone. The prepared Poloxamer 407 solution is mixed with BF Exo in a 9:1 ratio and the biodegradable zinc scaffold is immersed in the mixture at 4°C under vacuum conditions to ensure the complete filling of the scaffold with the BF Exo gel composite, resulting in the formation of the zinc scaffold/BF Exo composite implant (Figure 3A). Subsequently, the three-dimensional morphology of the biodegradable zinc scaffold/BF Exo composite implant is examined using scanning electron microscopy (Figure 3B). The images reveal a rough surface with numerous collapses and cracks at 50- and 200-times magnification, while at 1000- and 2000-times magnification, a small amount of exosomes is observed encapsulated within the gel. Furthermore, X-ray diffraction analysis of the zinc scaffold/BF Exo composite implant shows the highest intensity at a scanning angle of 20.0°, with lower intensity peaks around 43°, 54°, and 70°, indicating the coexistence of zinc and exosomes within the scaffold (Figure 3C). Moreover, after co-culturing with BMSCs for 3 days, the cell proliferation level is evaluated. The CCK-8 assay results demonstrate a significant increase in the cell proliferation level in the Zinc scaffold/BF Exo group compared to the Zinc scaffold group (Figure 3D). Additionally, the concentration of exosomal proteins in the zinc scaffold/BF Exo composite implant is found to increase with incubation time at 1, 3, 5, and 7 days (Figure 3E). Similarly, the zinc ion concentration in both the Zinc scaffold and Zinc scaffold/BF Exo groups also increases with incubation time, with the Zinc scaffold/BF Exo group showing a significantly reduced zinc ion release rate compared to the Zinc scaffold group (Figure 3F). These findings collectively indicate that loading exosomes onto the zinc scaffold leads to a deceleration in zinc ion release.

**Figure 4:**
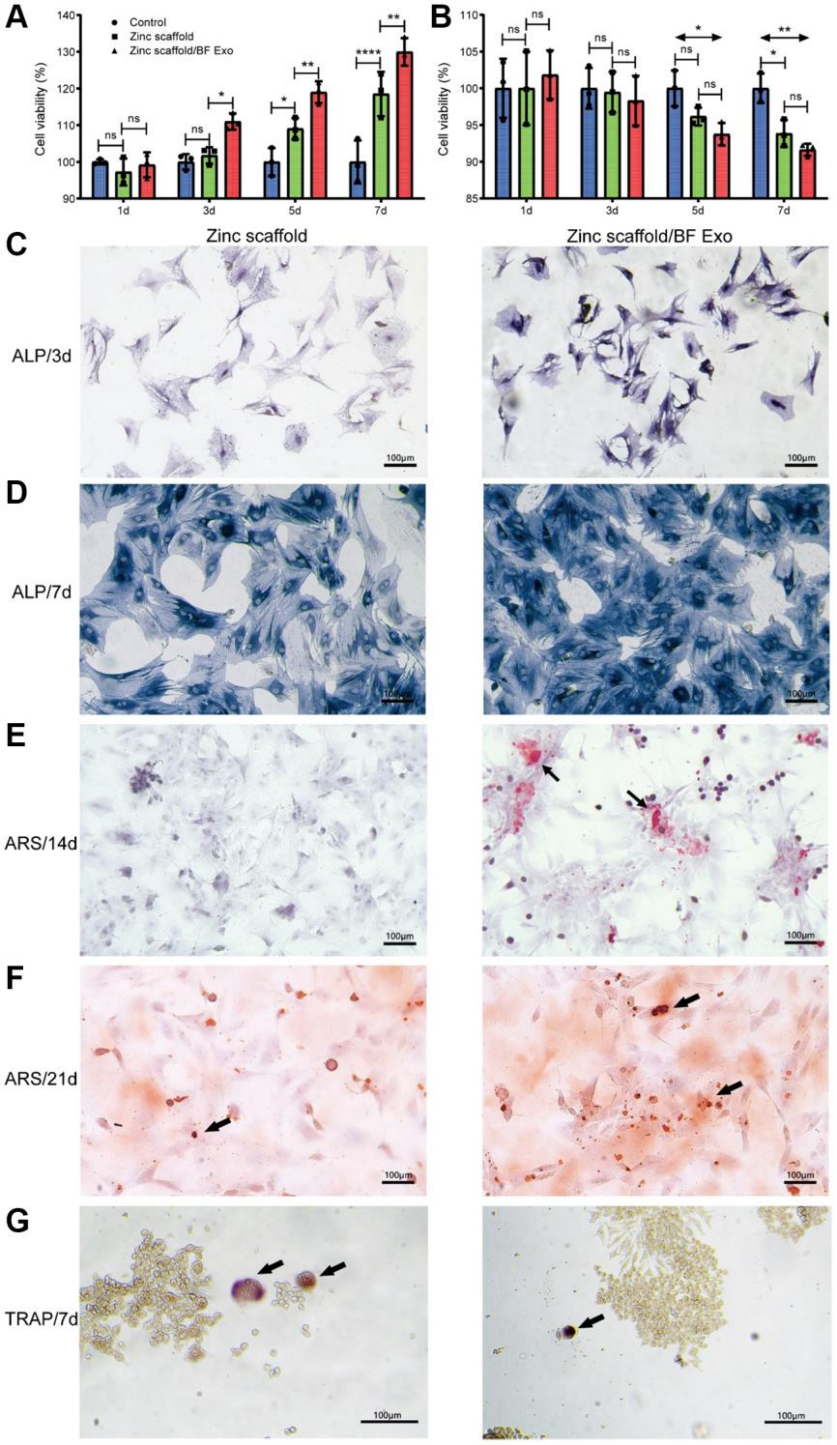
***In vitro* osteogenic performance assessment of zinc scaffold/BF Exo composite implant.** (**A**) The CCK-8 experiment was used to measure the cell viability of BMSCs after osteogenic induction for 1, 3, 5, and 7 days; (**B**) The CCK-8 experiment was used to assess the cell viability of RAW264.7 cells after osteoclast induction for the same time periods; (**C**, **D**) ALP staining was conducted for BMSCs after 3 and 7 days of osteogenic induction, where darker staining indicated stronger positive expression; (**E**, **F**) Alizarin Red staining was performed for BMSCs after 14 and 21 days of osteogenic induction; (**G**) TRAP staining was conducted for RAW264.7 cells after 7 days of osteoclast induction (^*^P < 0.05, ^**^P < 0.01, ^****^P < 0.0001, and ns for *P* > 0.05, *n* = 3).

Afterwards, this study conducted CCK-8 experiments to assess the cell viability of BMSCs for osteogenic induction and RAW264.7 cells for osteoclastic induction at 1, 3, 5, and 7 days post-treatment (Figure 4A, 4B). The results from the CCK-8 experiments on BMSCs for osteogenic induction showed that, compared to the control group, there were no significant differences in cell viability in the Zinc scaffold group at 1 and 3 days. However, at 5 and 7 days post-induction, the cell viability in the Zinc scaffold group notably increased. Additionally, at 1 day post osteogenic induction, the cell viability in the Zinc scaffold/BF Exo group showed no significant difference compared to the Zinc scaffold group. Conversely, at 3, 5, and 7 days post induction, the cell viability in the Zinc scaffold/BF Exo group significantly increased compared to the Zinc scaffold group (Figure 4A). Conversely, the results from the CCK-8 experiments on RAW264.7 cells for osteoclastic induction revealed that, compared to the control group, there were no significant differences in cell viability in the Zinc scaffold group at 1, 3, and 5 days. However, at 7 days post-osteoclastic induction, the cell viability in the Zinc scaffold group significantly decreased. Furthermore, compared to the Zinc scaffold group, there were no significant differences in cell viability in the Zinc scaffold/BF Exo group at 1, 3, 5, and 7 days post-osteoclastic induction. Nevertheless, at 5 and 7 days post-osteoclastic induction, the cell viability in the Zinc scaffold/BF Exo group significantly decreased compared to the control group, and its effects were even stronger than those of the Zinc scaffold group (Figure 4B).

After co-culturing the biodegradable zinc scaffold/BF Exo composite implant with BMSCs for 1 day, the culture medium was replaced with DMEM complete medium containing osteogenic induction fluid (with 10% FBS). Alkaline phosphatase staining was performed after osteogenic induction for 3 and 7 days, and Alizarin Red staining was conducted after 14 and 21 days to assess the *in vitro* osteogenic ability (Figure 4C–4F). Following osteogenic induction for 3 and 7 days, the results of alkaline phosphatase staining showed that the intensity of staining for BMSCs on Zinc scaffold and Zinc scaffold/BF Exo increased with the duration of osteogenic induction. In comparison to the Zinc scaffold group, the BMSCs on the Zinc scaffold/BF Exo exhibited deeper staining and stronger positive expression for alkaline phosphatase (Figure 4C, 4D). Furthermore, after 14 and 21 days of osteogenic induction, Alizarin Red staining revealed that the intensity of red mineralized nodules in BMSCs on both Zinc scaffold and Zinc scaffold/BF Exo increased with the duration of osteogenic induction. However, the BMSCs on the Zinc scaffold/BF Exo showed more defined calcium nodules compared to the Zinc scaffold group (Figure 4E, 4F). In addition, after 7 days of osteoclast induction, tartrate-resistant acid phosphatase (TRAP) staining of RAW264.7 cells revealed that osteoclasts exhibited multiple nuclei and were stained red (black arrows). The quantity of osteoclasts was reduced in the Zinc scaffold/BF Exo group compared to the Zinc scaffold group (Figure 4G). These findings collectively indicate that loading exosomes enhances the ability of the zinc scaffold to promote osteogenic cell activity and inhibit osteoclast activity.

### Assessment of *in vivo* osseointegration performance of zinc scaffold/BF Exo composite implants

**Figure 5:**
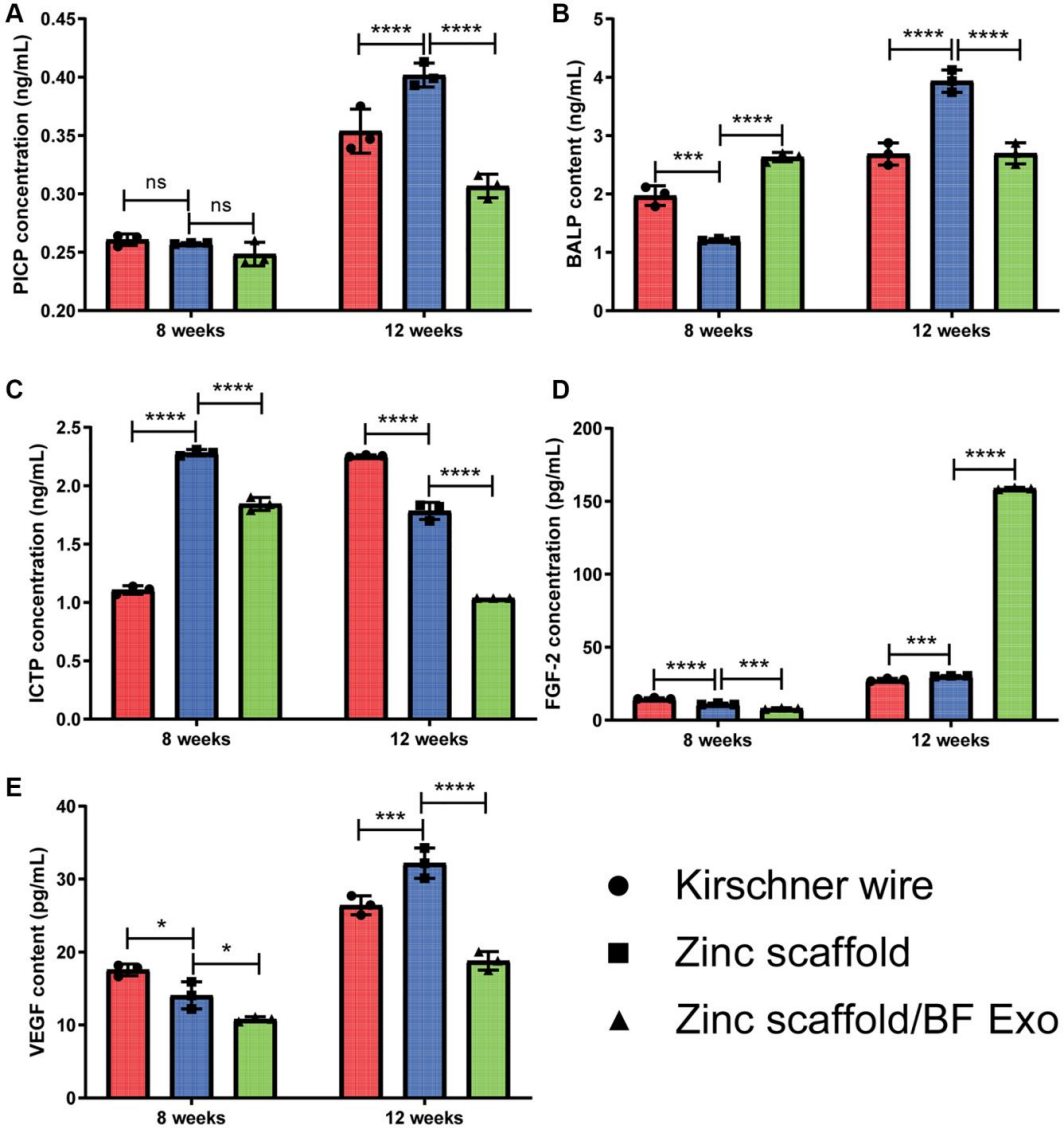
**The ELISA experiment on rabbit serum.** (**A**–**E**) The levels of PICP, BALP, ICTP, VEGF, and FGF-2 in the serum of rabbits were measured at 8 and 12 weeks after modeling (^*^P < 0.05, ^**^P < 0.01, ^****^P < 0.0001, ^*^P > 0.05, *n* = 3).

The study established a rabbit radius defect model and then divided the animals into groups to receive implantation of Kirschner wire, degradable zinc scaffold, and composite implantation of degradable zinc scaffold/highly bioactive serum exosomes (Zinc scaffold/BF Exo). Animals were sacrificed at 8 and 12 weeks post-implantation, and samples of rabbit blood, liver, kidney, and radius were collected. Furthermore, serum samples were collected from each group of New Zealand white rabbits at 8 and 12 weeks post-surgery, and ELISA was employed to assess the levels of bone formation markers (PICP and BALP), bone resorption marker (ICTP), and angiogenesis markers (VEGF, FGF2). The ELISA results at 8 weeks post-surgery analysis revealed that compared to the Kirschner wire group, the levels of BALP, VEGF, and FGF2 in rabbit serum from the Zinc scaffold group significantly decreased, while the ICTP content significantly increased. Additionally, compared to the Zinc scaffold group, the Zinc scaffold/BF Exo group showed a significant increase in BALP content, and significant decreases in ICTP, VEGF, and FGF2 levels (Figure 5A–5E). The ELISA results at 12 weeks post-surgery analysis demonstrated that, compared to the Kirschner wire group, the rabbit serum in the Zinc scaffold group exhibited significant increases in PICP, BALP, VEGF, and FGF2 levels, while the ICTP content significantly decreased. Furthermore, compared to the Zinc scaffold group, the rabbit serum in the Zinc scaffold/BF Exo group showed significant decreases in PICP, BALP, ICTP, and VEGF levels, and a significant increase in FGF2 content (Figure 5A–5E). These data indicate that in the early stage (8 weeks) of *in vivo* implantation experiments, zinc scaffold loaded with exosomes can enhance its ability to promote bone formation and suppress bone resorption, while in the late stage (12 weeks) of *in vivo* implantation experiments, loading exosomes can enhance the zinc scaffold's ability to suppress bone resorption and promote angiogenesis.

**Figure 6:**
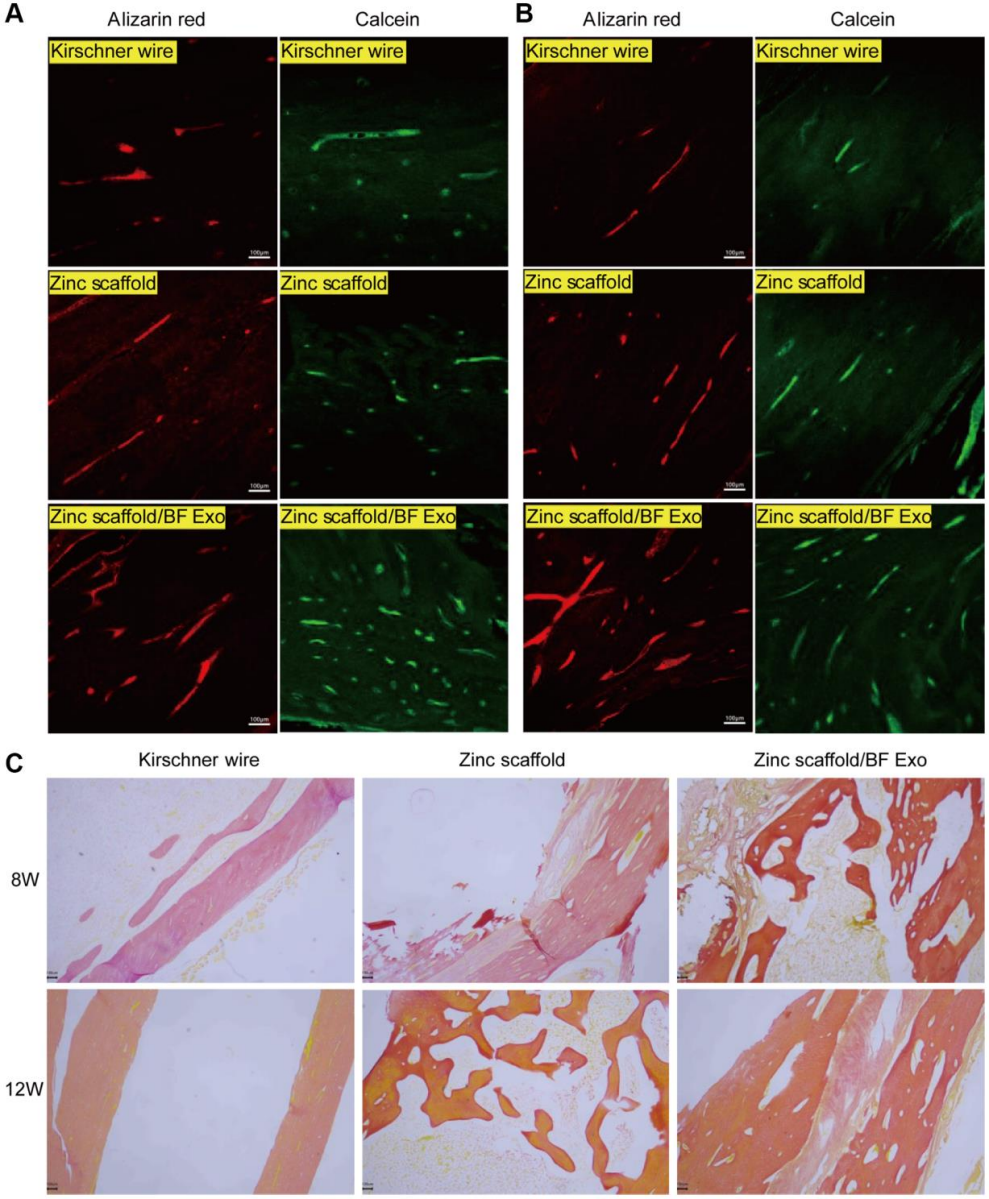
**Fluorescence and staining experiments on rabbit radius bones.** (**A**) Alizarin Red and Calcein Green fluorescence results of rabbit radius tissues after 8 weeks of modeling in each group; (**B**) Alizarin Red and Calcein Green fluorescence results of rabbit radius tissues after 12 weeks of modeling in each group; (**C**) Methylene blue-acidic magenta staining results of rabbit radius tissues after 8 and 12 weeks of modeling in each group (100×, *n* = 3).

Subsequently, in this study, fluorochrome labeling with Alizarin Red S and Calcein Green and Methylene Blue-Acid Magenta staining were performed on the rabbit radius bones from different groups of models to further evaluate the *in vivo* osteogenic performance of the Zinc scaffold/BF Exo composite implant. Under laser confocal microscopy, it was observed that at 8 and 12 weeks postoperatively, both the Zinc scaffold and Zinc scaffold/BF Exo groups demonstrated significant deposition of newly formed bone marked with Alizarin Red S and Calcein Green in comparison to the Kirschner wire group, with the latter showing more pronounced fluorescence (Figure 6A, 6B). Additionally, rabbits sacrificed at 8 and 12 weeks post-surgery underwent Methylene Blue-Acid Magenta staining. After removing the bone tissue from the scaffolds, decalcification and paraffin embedding were conducted for microscopic observation of the newly formed bone tissue. The results indicated that at 8 and 12 weeks post-surgery, the growth of newly formed bone tissue was faster in the Zinc scaffold group compared to the Kirschner wire group, and even faster in the Zinc scaffold/BF Exo group compared to the Zinc scaffold group (Figure 6C). These findings collectively demonstrate that the incorporation of extracellular vesicles can enhance the osteogenic effect of Zinc scaffolds in promoting new bone growth.

### Assessment of *in vivo* hepatorenal toxicity of zinc scaffold/BF Exo composite implant

**Figure 7:**
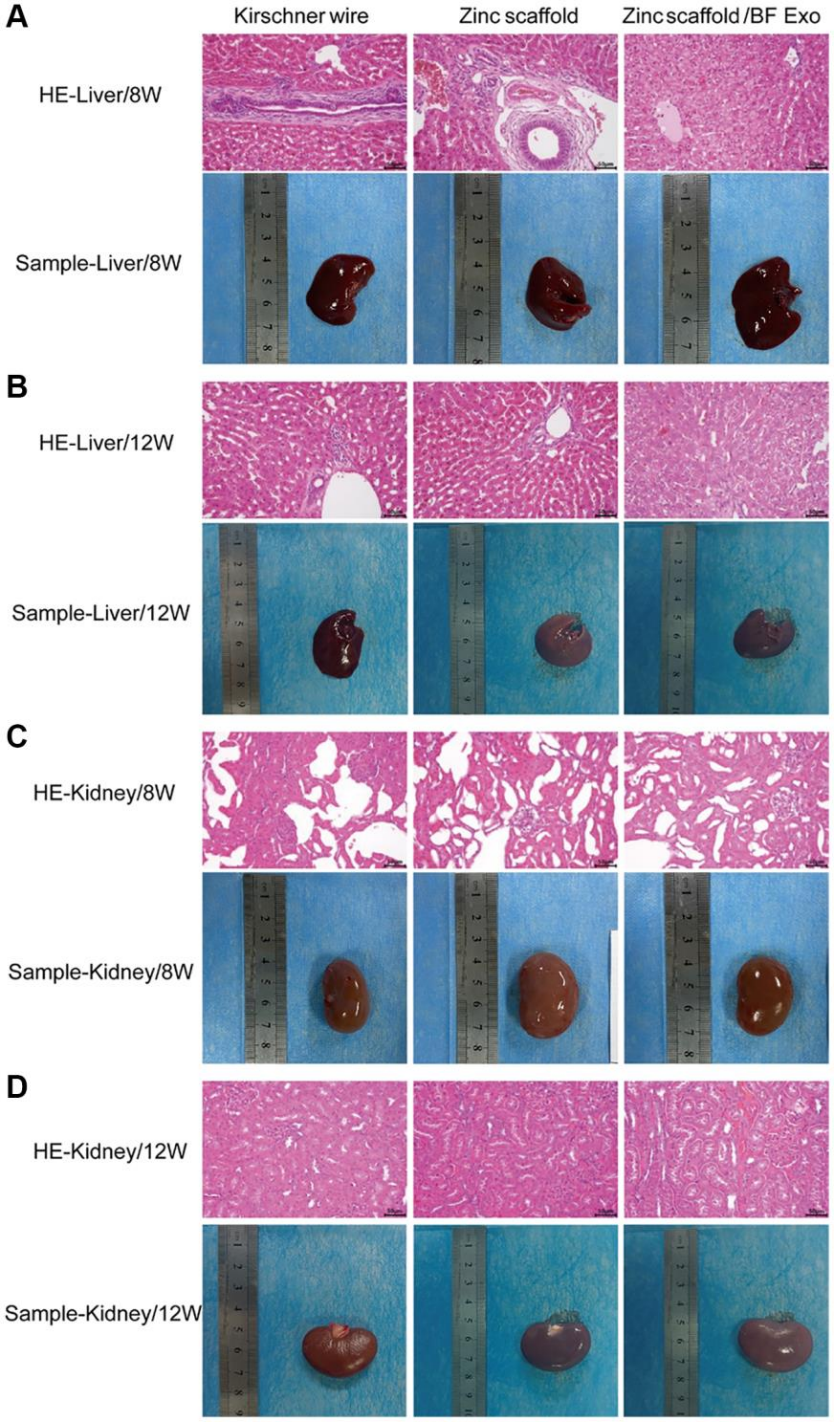
**Assessment of *in vivo* hepatorenal toxicity of zinc scaffold/BF Exo composite implant.** (**A**) HE staining results and physical samples of rabbit liver tissue after 8 weeks of modeling by each group; (**B**) HE staining results and physical samples of rabbit liver tissue after 12 weeks of modeling by each group; (**C**) HE staining results and physical samples of rabbit kidney tissue after 8 weeks of modeling by each group; (**D**) HE staining results and physical samples of rabbit kidney tissue after 12 weeks of modeling by each group (200×, *n*=3).

In order to evaluate the hepatic and renal toxicity of various implants on rabbits with radial bone defects, this study utilized HE staining to stain the liver and kidney tissues obtained from each group of rabbits. Subsequently, the stained tissues were observed under an optical microscope, and images were captured. After 8 and 12 weeks of modeling, severe interstitial hemorrhage and phenomena of fibrosis and inflammatory cell infiltration in the liver tissues were observed in the Kirschner wire group, Zinc scaffold group, and Zinc scaffold/BF Exo group compared to the Zinc scaffold group. Moreover, the phenomena of fibrosis and inflammatory cell infiltration in the liver were alleviated in the Zinc scaffold/BF Exo group when compared to the Zinc scaffold group. Furthermore, after 12 weeks of modeling, the phenomena of fibrosis and inflammatory cell infiltration in the liver were mitigated in the Kirschner wire group, Zinc scaffold group, and Zinc scaffold/BF Exo group compared to 8 weeks post modeling (Figure 7A, 7B). Additionally, in the kidney tissues of the Kirschner wire group, Zinc scaffold group, and Zinc scaffold/BF Exo group after 8 and 12 weeks of modeling, pathological conditions such as disordered renal tissue structure, glomerular atrophy, tubule dilation, and shedding of tubular epithelial cells were observed. Similar to the liver findings, the aforementioned pathological conditions were mitigated in the Zinc scaffold/BF Exo group compared to the Zinc scaffold group. Moreover, the pathological conditions mentioned above were alleviated in all groups after 12 weeks of modeling compared to 8 weeks post modeling (Figure 7C, 7D). These data indicate that the hepatic and renal toxicity of the zinc scaffold in rabbits is alleviated over time and that the loading of exosomes mitigates the hepatic and renal toxicity of the zinc scaffold.

### Micro-CT imaging experiment of *in vivo* implantation of zinc scaffold/BF Exo composite implant

**Figure 8:**
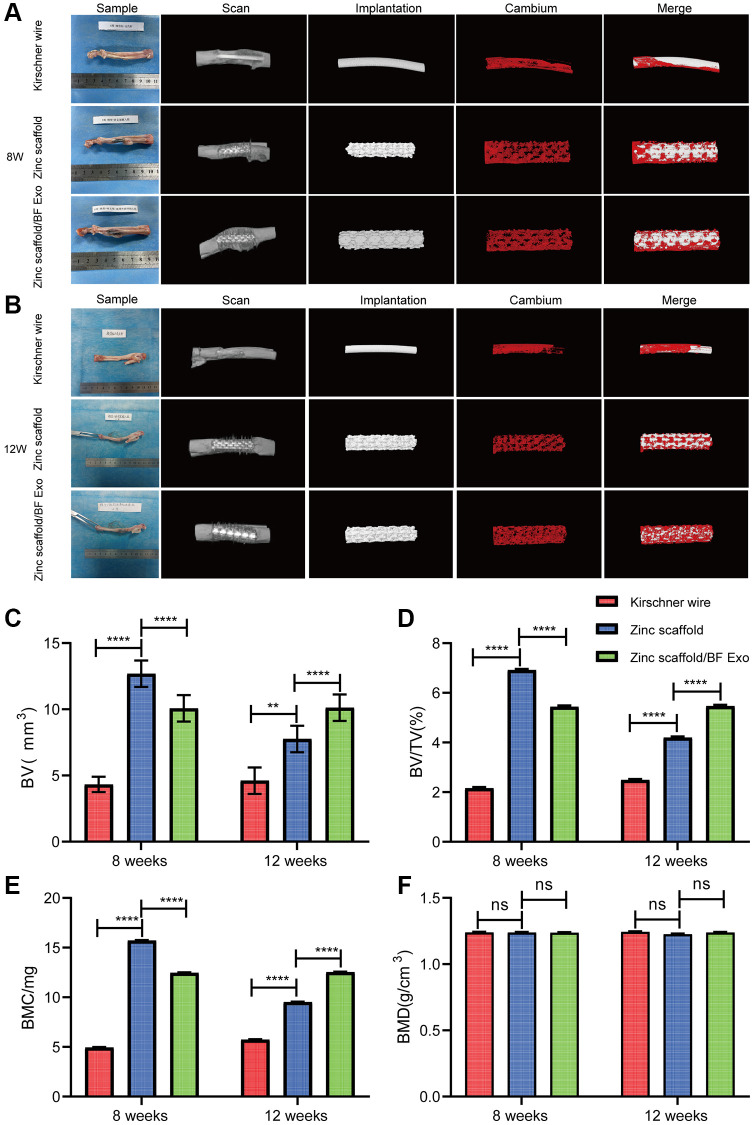
**Micro-CT imaging experiment of rabbit radius tissue.** (**A**) Imaging results of Micro-CT of rabbit radius in each group at 8 weeks after modeling. Contains Sample, Scan, Implantation, Cambium (New bone formation) and Merge images, respectively; (**B**) Imaging results of Micro-CT of rabbit radius in each group at 12 weeks after modeling; (**C**–**F**) Results of quantitative analyses of BV, BV/TV, BMC, and BMD in each group of animals at 8 and 12 weeks after modeling (^*^P < 0.05, ^**^P < 0.01, ^****^P < 0.0001, ^ns^*P* > 0.05 vs. Zinc scaffold, *n* = 3).

After fixation of all samples, Micro-CT (Germany, Bruker, SkyScan 1276) imaging experiments were conducted. The Micro-CT images of the rabbit radius at 8 and 12 weeks post-surgery for each group were displayed (Figure 8A, 8B). Additionally, quantitative analysis of the Micro-CT results was performed. The results indicated that at 8 weeks post-surgery, compared to the Kirschner wire group, the Zinc scaffold group showed increased BV, BV/TV, and BMC, while BMD did not exhibit significant changes. When compared to the Zinc scaffold group, the Zinc scaffold/BF Exo group showed decreased BV, BV/TV, and BMC, with no significant change in bone mineral density (BMD) (Figure 8C–8F). After 12 weeks, in comparison to the Kirschner wire group, the Zinc scaffold group showed increased BV, BV/TV, and BMC, with no significant change in bone mineral density (BMD). When compared to the Zinc scaffold group, the Zinc scaffold/BF Exo group exhibited increased BV, BV/TV, and BMC, while BMD did not significantly change (Figure 8C–8F). These data indicate that the zinc scaffold facilitates healing of rabbit radius defects. The early (8 weeks) application of zinc scaffold is associated with a diminished healing effect when loaded with highly bioactive serum exosomes, while the late stage (12 weeks) demonstrates an enhanced healing effect of the zinc scaffold.

### Immunohistochemical analysis of *in vivo* implantation of zinc scaffold/BF Exo composite implant

**Figure 9:**
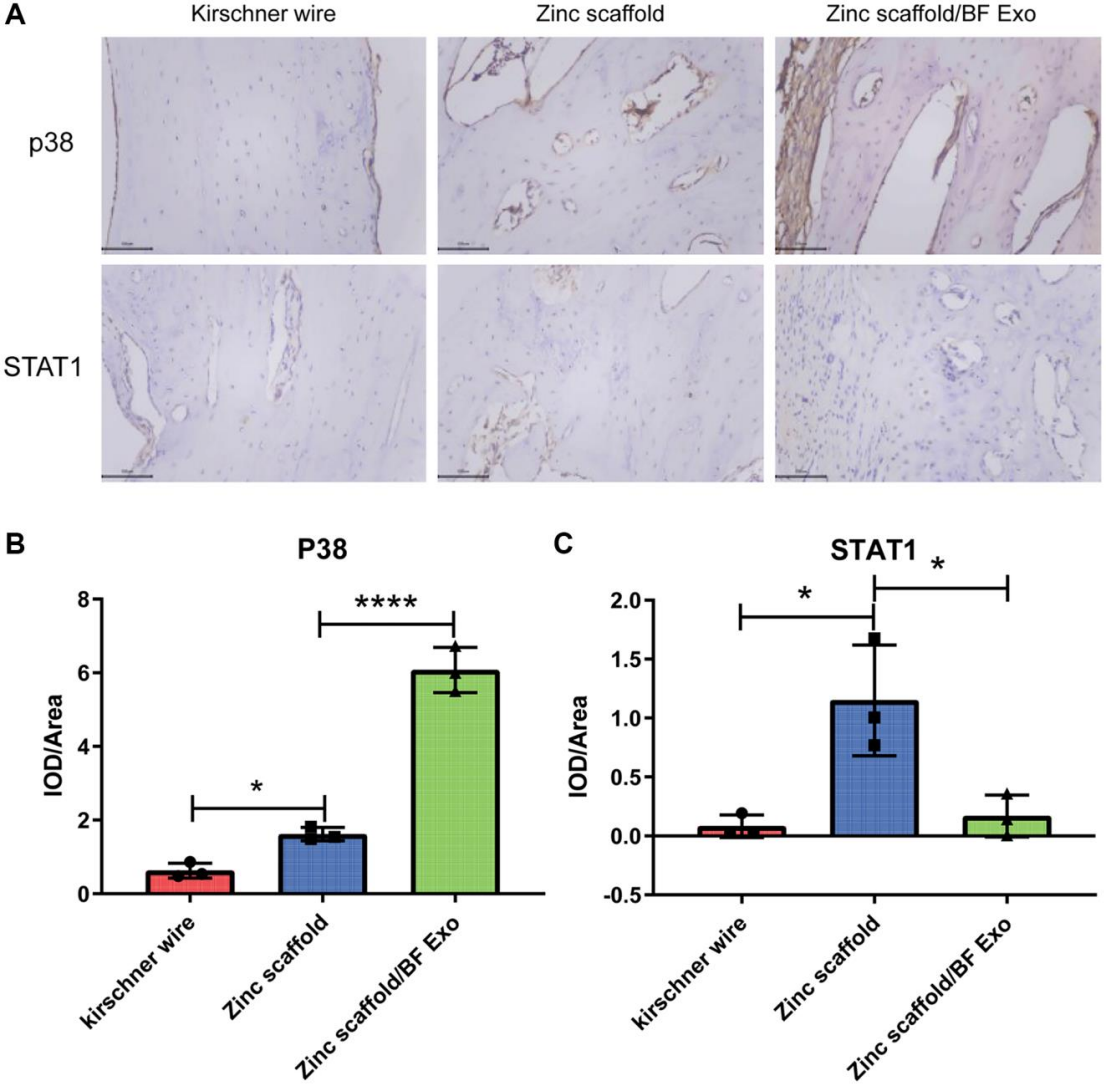
**Immunohistochemical analysis of *in vivo* implantation of zinc scaffold/BF Exo composite implant.** (**A**) Immunohistochemical staining results of rabbit radius in each group after 12 weeks of modeling; (**B**) Quantitative analysis results of p38 in rabbit radius in each group after 12 weeks of modeling; (**C**) Quantitative analysis results of STAT1 in rabbit radius in each group after 12 weeks of modeling (^*^P < 0.05, ^****^P < 0.0001, ^ns^*P* > 0.05 vs. Zinc scaffold, *n* = 3).

Next, this study used immunohistochemical analysis to detect the protein expression levels of p38 and STAT1 in the bone tissue of New Zealand white rabbits in all groups 12 weeks post-surgery (Figure 9A). The results of the quantitative analysis indicated a significant increase in the expression of p38 and STAT1 in the Zinc scaffold group compared to the Kirschner wire group. Additionally, compared to the Zinc scaffold group, the Zinc scaffold/BF Exo group showed a significant increase in p38 expression and a significant decrease in STAT1 expression (Figure 9B, 9C). These data suggest that in the late stage of fracture healing (12 weeks), the zinc scaffold can promote the expression of p38 and STAT1. Moreover, loading with high bioactive serum exosomes promotes the expression of p38 while inhibiting the expression of STAT1.

## DISCUSSION

Currently, the repair of large bone defects faces significant challenges such as substantial bone regeneration and vascular reconstruction [26]. Previous studies have demonstrated that the addition of magnesium or zinc can improve the mechanical performance and osteoinductivity of scaffolds [4]. However, the rapid degradation of magnesium makes zinc a more suitable metal for use in degradable bone grafts. Nevertheless, a limitation of zinc-containing scaffolds is the toxicity resulting from the excessive release of zinc ions [7]. In this study, a porous zinc scaffold was prepared using laser melting rapid prototyping 3D printing technology. The scaffold exhibits interconnected internal pores with a large pore size of approximately 500 micrometers and a porosity of 60–70%. This multi-level and porous structure, resembling natural cancellous bone, facilitates the infiltration, proliferation, and osteogenic differentiation of BMSCs, as well as enhances the inward growth of bone tissue to improve the integration of bone in the body [16, 28]. Concurrently, zinc ions, as an essential trace element, play a crucial role in improving the cellular immune environment, accelerating bone regeneration, and inhibiting biofilm formation [29]. Furthermore, Poloxamers, a type of thermosensitive polymer, possess good solubility, low toxicity, strong drug release capability, and good compatibility with numerous biological molecules and chemical excipients [30]. Specifically, Poloxamers 407 have been widely used in the delivery of drugs and biological molecules through different routes [31]. Therefore, this study designed a bifunctional exosome loaded with Poloxamer 407 as a base, which is beneficial for the *in situ*-controlled release of bifunctional exosomes and promotes bone and vascular regeneration [26, 32]. The successful mixing of serum exosomes and Poloxamer 407 hydrogel, as well as their uniform dispersion within the degradable zinc scaffold, was demonstrated. The FTIR results indicate that the exosomes do not undergo chemical reactions with Poloxamer 407 or the degradable zinc scaffold, as no characteristic peaks were detected.

Subsequently, this study conducted MTT experiments by co-culturing L-929 cells with the extraction of degradable porous zinc scaffolds. The results indicated no significant difference in cell viability between the degradable 3D-printed porous zinc scaffold immersion group and the control group. This finding may be related to the scaffold's porous structure and the relatively short co-cultivation time. It is therefore necessary for future research to extend the co-cultivation time, increase scaffold surface coatings, and manufacture various alloy scaffolds to evaluate the cytotoxicity of implants [10, 28]. Additionally, in order to assess the hepatic and renal toxicity of the implanted materials in the model rabbits, this study used HE staining to stain the liver and kidney tissues obtained from each group of rabbits. The results demonstrated that the hepatic and renal toxicity of zinc scaffolds was alleviated as time progressed, and the loading of exosomes reduced the hepatic and renal toxicity of zinc scaffolds. This may be attributed to the controlled release of Poloxamer 407 hydrogel [26]. Therefore, these findings indicate that the degradable porous zinc scaffolds designed in this study exhibited no significant cytotoxicity to cells and hepatic and renal tissues.

In addition, this study utilized CCK-8 experiments to assess the cell viability of BMSCs following osteogenic induction and RAW264.7 cells following osteoclastogenic induction. Alkaline phosphatase staining was employed to evaluate the osteoinductivity of the scaffolds, while Alizarin Red staining was used to observe the degree of osteogenic differentiation. Furthermore, tartrate-resistant acid phosphatase staining was utilized to characterize osteoclastic activity [33, 34]. The results demonstrate that exosome loading enhances the zinc scaffold's ability to promote osteogenic cell activity and suppress osteoclastic activity. Previous studies have indicated that zinc can stimulate the Wnt/β-catenin and NF-κB signaling pathways to promote osteogenic cell proliferation, differentiation, and collagen synthesis [35, 36]. Although exogenous bone grafts can lead to prolonged M1 macrophage polarization, creating an inflammatory bone microenvironment that hinders bone defect repair [27, 37], the presence of BF exosomes may reduce the number of M1 macrophages and promote vascular and bone formation [26].

Due to the current lack of research on the mechanism of zinc-containing biomaterials to promote bone repair, analyzing the expression of genes and proteins related to osteogenesis, osteoclastogenesis, and angiogenesis at different stages of bone defect repair can help to explore the potential molecular mechanisms of degradable zinc scaffolds/BF Exo composite implants. Bone-specific alkaline phosphatase (BALP) is an extracellular enzyme of osteoblasts, serving as a marker of osteoblast maturation and activity [38]. I-type procollagen carboxy-terminal peptide (PICP) and I-type procollagen amino-terminal peptide (PINP) levels in the serum reflect the capacity of osteoblasts to synthesize bone collagen and can be used to monitor osteoblast activity and bone formation, providing specific and sensitive indicators of new bone formation [39, 40]. The carboxy-terminal telopeptide of type 1 collagen (CTX-1) is released into the blood during bone resorption as a result of the degradation of type 1 collagen molecules, serving as a specific reflection of the level of bone resorption [39]. Vascular endothelial growth factor (VEGF) and fibroblast growth factor 2 (FGF-2) are potent angiogenic activators that stimulate the migration and proliferation of endothelial cells in existing blood vessels, thereby promoting the generation and stabilization of new blood vessels [41–43]. In this study, compared to the Zinc scaffold group, the Zinc scaffold/BF Exo group showed a significant increase in BALP content and a significant decrease in ICTP, VEGF, and FGF2 content at 8 weeks postoperatively. At 12 weeks postoperatively, the Zinc scaffold/BF Exo group exhibited a significant decrease in PICP, BALP, ICTP, and VEGF content in rabbit serum, while FGF2 content significantly increased. The ELISA experimental data of this study indicated that early (8 weeks) *in vivo* implantation experiments with zinc scaffold-loaded exosomes enhanced the ability of the zinc scaffold to promote osteogenesis and inhibit osteoclastogenesis, whereas late (12 weeks) *in vivo* implantation experiments loaded with exosomes enhanced the ability of the zinc scaffold to inhibit osteoclastogenesis and promote angiogenesis.

Additionally, the findings were further supported by the experiments involving Alizarin Red S and Calcein Green fluorochrome labeling, Methylene Blue-Acid Magenta staining, and Micro-CT imaging. Alizarin Red staining, commonly used to visualize the degree of osteogenic differentiation [33, 44], indicated a significant increase in new bone deposition in both Zinc scaffold and Zinc scaffold/BF Exo groups compared to the Kirschner wire group, as evidenced by intense red coloration. Calcein Green fluorochrome labeling was used to assess the toxicity of the materials and indicated a higher number of viable cells in both experimental groups [45, 46]. Additionally, Methylene Blue-Acid Magenta staining displayed clear visualization of osteoblasts, extracellular matrix, and osteoid, aiding in the characterization of static bone formation parameters [47]. Micro-CT imaging revealed a substantial increase in bone volume, bone volume/total volume ratio, and bone mineral content in both experimental groups compared to the Kirschner wire group, with more pronounced enhancements observed in the Zinc scaffold/BF Exo group. However, it also indicated a temporary weakening of the zinc scaffold’s osteogenic potential at an early stage (8 weeks) after BF Exo loading, possibly due to the controlled release of BF Exo by Poloxamer 407 and its inhibitory effect on the zinc scaffold's release rate. Nevertheless, the later stages (12 weeks) showed a significant improvement in the zinc scaffold's osteogenic potential post-BF Exo loading. These results suggest the need for further optimization of the composition and structure of hydrogels to modulate the controlled release of exosomes or drugs.

Finally, this study employed immunohistochemistry to investigate the potential mechanisms underlying the promotion of bone defect repair by degradable zinc scaffolds/BF Exo composite implants. The quantitative analysis from the immunohistochemistry revealed that at the late stage of fracture healing (12 weeks), the zinc scaffold could enhance the expression of p38 and STAT1. Further, loading with highly bioactive serum exosomes was found to further promote the expression of p38 while inhibiting the expression of STAT1. The p38 mitogen-activated protein kinase (MAPK) pathway plays a crucial role in the response of osteoblasts to various osteogenic ligands during osteoblast differentiation, extracellular matrix deposition, and mineralization processes [48]. Previous research has demonstrated that irisin can promote osteoblast proliferation and differentiation by increasing the phosphorylation of the p38/ERK MAPK signaling pathway [49]. Additionally, Signal Transducer and Activator of Transcription 1 (STAT1) is a member of the STAT protein family and can activate signaling pathways involved in various physiological and pathological responses [50]. Multiple studies have indicated that STAT1 can induce cellular apoptosis and hinder fracture repair [51, 52]. In conclusion, the degradable zinc scaffold/BF Exo composite implants developed in this study enhance the osteogenic, anti-osteoclastic, and pro-angiogenic capabilities during bone defect repair, which may be associated with the regulation of p38/STAT1 by BF Exo. However, the specific regulatory mechanisms require further investigation.

The combination of porous zinc scaffolds with exosomes and hydrogels provides a new extracellular signaling pathway and therapeutic platform, featuring a composite material with both mechanical support and bioactive signals. Integrating bioactive exosomes with 3D-printed porous zinc scaffolds leverages the bioactivity of the exosomes and the structural characteristics of the zinc scaffolds to provide a dual mechanism for bone regeneration. This combination improves osteogenic efficiency while ensuring that the scaffold structure is closer to the natural bone microenvironment, providing an excellent platform for new bone growth and vascular invasion. The introduction of Poloxamer 407 thermosensitive gel enhances the biomaterial's functionality and biocompatibility, offering a material that transitions from liquid to gel state at body temperature to fill defect areas and achieve controlled release of bioactive substances for sustained and stable release. This represents a new attempt at bone defect repair. The combined treatment strategy integrating physical support (porous zinc scaffold), drug therapy (drug-loaded gel), and biological therapy (exosomes) demonstrates a multidimensional effect in treating bone defects. This strategy provides a new method for treating bone defects, potentially offering better biocompatibility and osteogenic efficiency than traditional autologous or allogeneic bone grafts and reducing the risk of secondary surgeries. The long-term stability and biosafety assessment of exosomes are crucial for clinical translation. Further research is needed to ensure these therapeutic approaches are safe for patients. Although significant progress has been made in the fields of materials science and biomedical engineering, subsequent studies are needed to improve existing materials and methods. Future research should delve deeper into these issues to enhance the effectiveness and safety of bone defect repair materials and methods and to further explore their underlying mechanisms.

## CONCLUSION

The study at hand utilized laser melting rapid prototyping 3D printing technology to fabricate a porous zinc scaffold, and in combination with serum exosomes rich in bone-forming factors and poloxamer 407 thermosensitive hydrogel under vacuum conditions, a novel bone repair implant was obtained. The biodegradable porous zinc scaffold/BF Exo composite implant achieved enhanced osteogenesis and bone resorption inhibition in the early stage of bone repair through the combined release of zinc and BF Exo, followed by sustained bone conduction and induction through the continuous release of zinc. In addition, the loading of BF Exo enhanced the bone resorption inhibition and promotion of angiogenesis capabilities during bone defect repair, which may be related to the regulation of p38/STAT1 by BF Exo. The combined treatment of porous zinc scaffold and BF Exo is an effective and biocompatible strategy for bone defect repair. Further improvement of the composition of the porous zinc scaffold should be pursued, along with further adjustment of the safe and effective degradation rate of the zinc scaffold. Integration of exosomes with diverse efficacies for the synthesis of specifically functionalized composite materials can be explored for the treatment of various clinical diseases.

## Supplementary Materials

Supplementary Figure 1
