# Supplementary material for: 3D-printed porous zinc scaffold combined with bioactive serum exosomes promotes bone defect repair in rabbit radius
Source: Aging (Albany NY). 2024 May 31;16(11):9625–48. doi: 10.18632/aging.205891 (PMC11210218; doi:10.18632/aging.205891)
Supplement: Supplementary Figure 1 [file aging-16-205891-s001.pdf]

SUPPLEMENTARY FIGURE

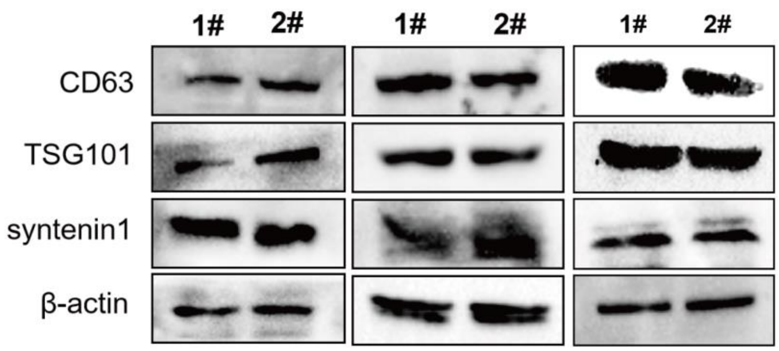

Supplementary Figure 1. Illustrates the protein expression of CD63, TSG101, and syntenin1 in exosomes from the serum of rabbit models with femoral fractures.
